# A Time-Based Meta-Analysis on the Incidence of New Onset Diabetes after Liver Transplantation

**DOI:** 10.3390/jcm10051045

**Published:** 2021-03-03

**Authors:** Yip Han Chin, Hon Qin Marcus Tan, Cheng Han Ng, Darren Jun Hao Tan, Snow Yunni Lin, Daniel Q. Huang, Chin Meng Khoo, Mark Dhinesh Muthiah

**Affiliations:** 1 Yong Loo Lin School of Medicine, National University of Singapore, Singapore 117597, Singapore; c.yiphan@u.nus.edu (Y.H.C.); e0360761@u.nus.edu (H.Q.M.T.); e0433903@u.nus.edu (D.J.H.T.); e0416073@u.nus.edu (S.Y.L.); daniel_huang@nuhs.edu.sg (D.Q.H.); mdckcm@nus.edu.sg (C.M.K.); 2 Department of Medicine, National University Hospital, Singapore 119074, Singapore; 3 National University Centre for Organ Transplantation, National University Hospital, Singapore 119074, Singapore

**Keywords:** NODAT, liver transplantation, new onset diabetes after transplantation, type 2 diabetes, incidence

## Abstract

NODAT (new-onset diabetes after transplantation) is an important complication after liver transplant, however, there is variation in the reported incidence of NODAT. Therefore, a meta-analysis was performed to estimate the incidence of NODAT in liver transplant. Electronic databases were searched for articles regarding NODAT incidence after liver transplantation. Incidence of NODAT were analyzed at six different timepoints. Summary statistics were calculated using a generalized linear mixed model in random effects. 28 articles were included and out of a pooled population of 71,257 patients, overall incidence of NODAT was found to be 15.51%, 16.09%, 18.30%, 20.86%, 18.08%, 25.05% for three-months, six-months, one-year, three-year, five-year, and ten-year timepoints respectively. After a sensitivity analysis which only included articles with clear definitions of NODAT, the incidence of NODAT was found to be higher at three-year (21.79%), five-year (25.82%), and ten-year (44.95%) timepoints. Subgroup analysis according to ethnicity found no significant differences for all timepoints. However, studies with predominantly Asian participants generally had a higher incidence of NODAT. In conclusion, this meta-analysis provides a pooled estimate of the incidence of NODAT following liver transplantation. Further studies are required to provide a more comprehensive understanding on how ethnicity can affect the incidence of NODAT.

## 1. Introduction

Liver transplantation is the only definitive treatment for end stage liver disease, and one of the only curative treatment options for hepatocellular carcinoma (HCC) [1]. New-onset diabetes after transplantation (NODAT) is a common and important complication estimated to occur in 15–30% of recipients who were not known to be diabetic before the transplant [2]. It is also associated with an increased risk of early mortality [3], major cardiovascular events [4], renal impairment [5], biliary strictures [6], rejection episodes [7], and graft loss [8]. Despite its prevalence and important effects on patient outcomes, an international consensus guideline was only established in 2003 by the World Health Organization (WHO) following the American Diabetes Association (ADA) methods for diagnosing diabetes [9].

Risk factors for NODAT after liver transplant can generally be classified into non-modifiable and modifiable factors [10]. Non-modifiable factors which increase the risk of NODAT include host factors such as older age [11], family history of DM, ethnicity [11], underlying diseases such as hepatitis C virus (HCV) infection and liver cirrhosis [6,12,13], and donor factors such as donor age (> 60 years old) [14], presence of liver steatosis in donor livers [15], and male donors [6]. While modifiable factors which raise the risk of NODAT may include the type of immunosuppressive regimens utilised, such as corticosteroids or tacrolimus-containing regimens [11,12], high body mass index (BMI) [12] and length of intensive care unit (ICU) stay [6]. The effects of these in relations to genetics and ethnicity have also been brought into attention in recent years [16]. Hence, better identification of these risk factors and how it affects the incidence of NODAT in countries may prove crucial to improve the outcomes of liver transplant patients.

While the incidence and risk factors of NODAT in recipients of kidney transplants have been well established [17], the incidence of NODAT in liver transplant recipients is less clear, with several studies reporting heterogenous results due to differing definitions, length of follow-up and diagnostic criteria [2,10,18]. A previous meta-analysis has been conducted by Heisel et al. in 2003 [19]. However, this was before the international consensus by the WHO and ADA guidelines and this would have led to increased variations in the threshold for the diagnosis and reporting of NODAT cases in their included articles [19]. Thus, this meta-analysis was conducted to estimate the pooled incidence of NODAT after liver transplantation, adjusting for the different definitions of NODAT and analysing the incidence at predefined timepoints.

## 2. Materials and Methods

### 2.1. Search Strategy

Taking guidance from the Meta-Analysis of Observational Studies in Epidemiology (MOOSE) [20] and PRISMA [21], electronic databases (Medline and Embase) were searched for articles examining the incidence of NODAT after liver transplantation from inception till August 2020 and downloaded into EndnoteX9. Key search terms such as “incidence”, “new-onset diabetes” and “liver transplantation” were used in the search strategy. Manual sieving of the references of the included articles was also conducted, and duplicate studies were removed using Endnote X9. The search was done in consultation with a medical librarian and the full search strategy can be found in the Supplementary Material 1.

### 2.2. Inclusion Criteria and Data Extraction

Two authors (YHC, HQMT) independently screened all articles retrieved from the search, and articles that met the inclusion criteria were marked for inclusion. Any discrepancies were resolved in consultation with an independent third author (CHN). Inclusion criteria included: (1) articles that studied the incidence of NODAT in adult patients after liver transplantation, (2) original articles that were translated into or written in the English language, (3) articles examining the incidence of NODAT at specific timepoints after liver transplantation, namely: three-months, six-months, one-year, three-year, five-year and ten-year timepoints. Exclusion criteria included: (1) articles that studied the incidence of NODAT following other forms of organ transplant such as kidney transplant, (2) articles that included pediatric populations, (3) articles that examined NODAT from the same database or hospital centers with overlapping time periods, and (4) dual organ recipients (heart and liver transplant, kidney and liver transplant, etc.).

Key data such as patient demographics (BMI, age and gender), characteristics of included articles (sample size, country, follow-up time), definition of NODAT (WHO and ADA), postoperative medications (tacrolimus, steroids) and incidence of NODAT at the intervals previously mentioned were extracted by two authors (YHC, HQMT) independently into a predefined datasheet. Any discrepancy was resolved by discussion with a third author (CHN). Manual extraction of the incidence of NODAT from the Kaplan–Meier curves using WebPlotDigitalizer was used when raw numbers were not available from the articles. Estimated values of the mean and standard deviation were derived through formulas when they were not provided [22].

### 2.3. Statistical Analysis and Quality Assessment

All analyses were conducted using R (RStudio 1.3.1073). Pooled proportions of the incidence of NODAT after liver transplantation were analyzed at the intervals previously mentioned. Analysis of proportions was undertaken using a generalized linear mixed model (GLMM) instead of Freeman–Tukey double arcsine transformation as GLMM has been shown to be the most accurate method for transformation for meta-analysis of single proportions in numerous simulation studies [23]. No continuity correction was applied and all analyses were conducted in random effects regardless of heterogenicity measures (I^2^, tau, Cochran Q test) [23]. As single arm meta-analysis data are usually heterogenous, single-arm prevalence reviews often show substantial heterogeneity when the interpretation of I^2^ alone is used in the quantification of heterogeneity [24,25]. I^2^ can be especially misleading in large datasets as I^2^ increases with sample size [26].

A subsequent sensitivity analysis was conducted to include only articles using WHO and ADA criteria for NODAT for a more homogenous analysis. In addition, further subgroup analysis was also conducted based on the predominant ethnicity of the patients in the included study, where articles were classified into articles with predominantly Asian patients and articles with predominantly Western patients [27,28]. Analysis of publication bias was also conducted through visual inspection of funnel plots when sufficient studies were available (*n* > 9) [29]. Quality assessment of cohort studies was conducted via the Newcastle Ottawa Scale (NOS), which evaluates the quality of the articles using three criteria including selection, comparability and outcome [30].

## 3. Results

### 3.1. Search Results and Study Characteristics

**Figure 1:**
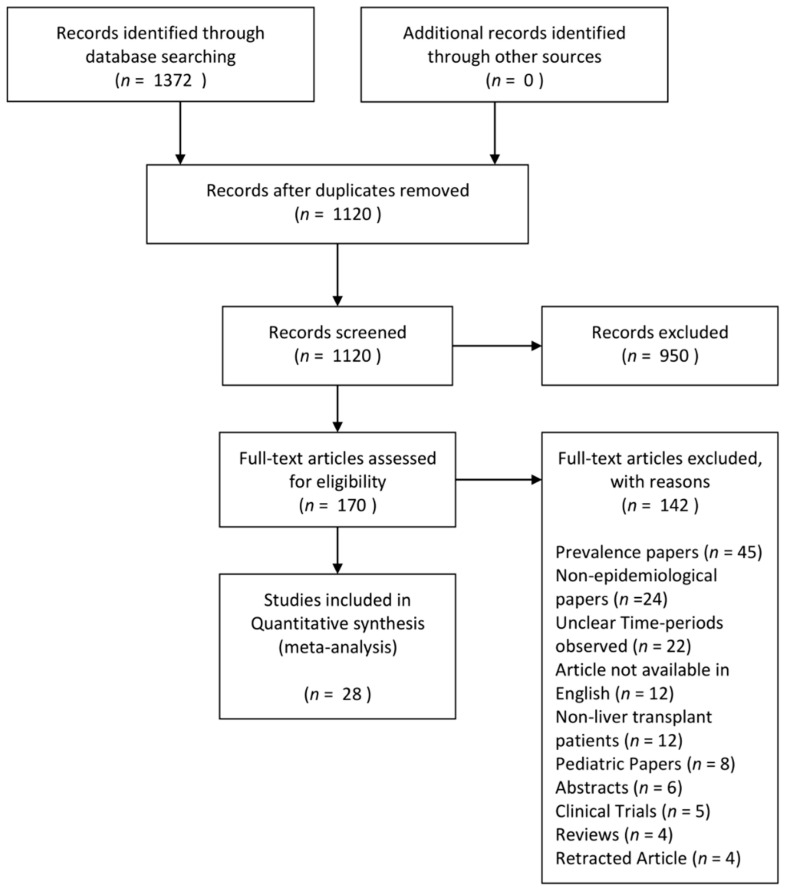
Flow chart of systematic literature search and screening for incidence of New onset diabetes after liver transplantation.

1372. articles were identified after the search, and 170 articles were selected for full text review, of which 28 met the inclusion criteria. Figure 1 shows the flowchart of the inclusion process. The articles originated from various countries, with six articles originating from each USA [31,32,33,34,35,36] and China [6,15,37,38,39] respectively, three from France [40,41,42], two each from India [43,44], Japan [45,46], Spain [47,48], one each from Canada [49], Egypt [50], Germany [51], Iran [52], Korea [53], Taiwan [54], UK [55]. Studies were conducted from 1989 till 2018. A total of 71,257 patients were included in the various studies and the mean age of the participants ranged from 39.24 to 55.61 years old. Quality assessment of included articles was conducted, and most articles were of moderate to high quality. The details of the quality assessment and characteristics of the included articles are presented in Table 1, and articles included in this article can be seen in the Supplementary References list.

### 3.2. Definition of NODAT

Definition of NODAT was based on the 2003 WHO and ADA criteria [9], defined as a fasting glucose level of ≥7.0 mmol/L, or a non-fasting glucose level of ≥11.1 mmol/L confirmed on at least two occasions or a need for antidiabetic drugs after the first post-transplant month [9]. Nine articles defined NODAT without reference to the WHO and ADA criteria, and 19 articles followed the WHO and ADA guidelines for NODAT after liver transplantation.

### 3.3. Overall Incidence of NODAT

**Figure 2:**
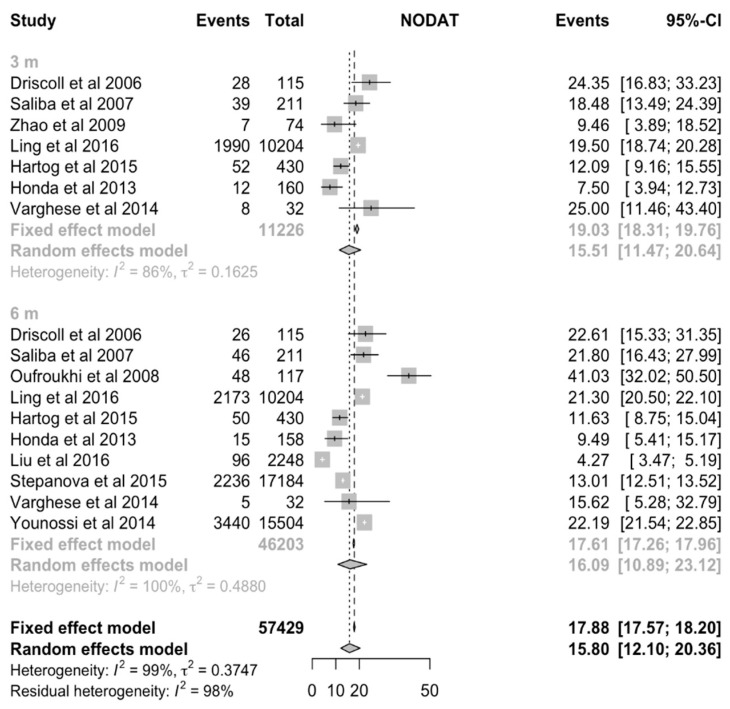
Incidence of New-Onset Diabetes after Transplantation (NODAT) at 3-months and 6-months after Liver Transplantation. CI = Confidence interval.

**Figure 3:**
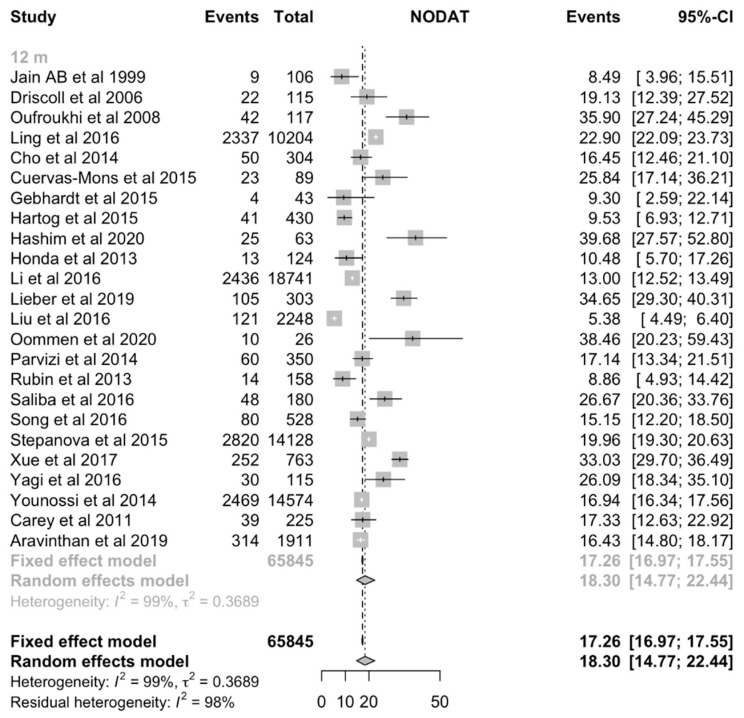
Incidence of New-Onset Diabetes after Transplantation (NODAT) at 1-year after Liver Transplantation. CI = Confidence interval.

**Figure 4:**
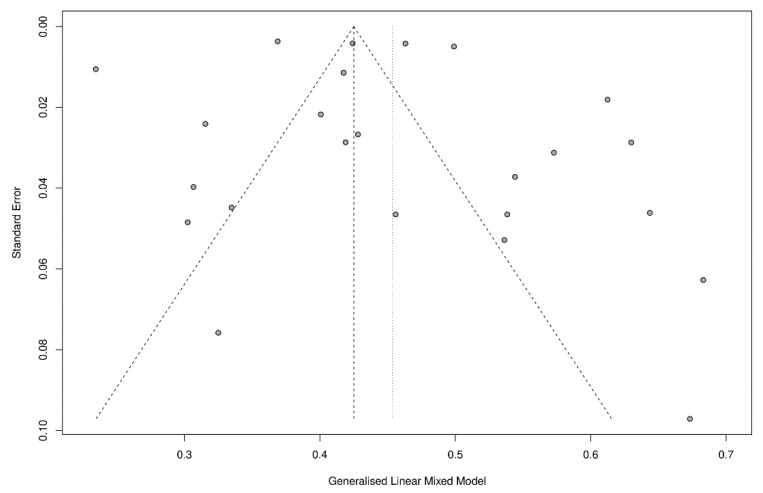
Funnel Plot of Incidence of New-Onset Diabetes after Transplantation (NODAT) at 1-year after Liver Transplantation.

The incidence of NODAT after liver transplantation was analyzed at three-months, six-months, one-year, three-year, five-year and ten-year timepoints. The overall incidence of NODAT after liver transplantation was found to range between 15% to 25% (Table 2). The pooled incidence of NODAT was 15.5% (CI:11.5% to 20.7%, Figure 2), 16.1% (CI:10.9% to 23.1%, Figure 2), 18.3% (CI:14.8% to 22.4%, Figure 3), 20.9% (CI:13.0% to 31.8%), 18.08% (CI:10.3% to 29.9%), 25.05% (CI:11.2% to 47.1%) for three-months, six-months, one-year, three-year, five-year and ten-year timepoints respectively (Table 2). Visual inspection of funnel plot suggests significant publication bias for 1-year (Figure 4), 6-months, three-years, and five-years timepoints (Supplementary Material).

**Figure 5:**
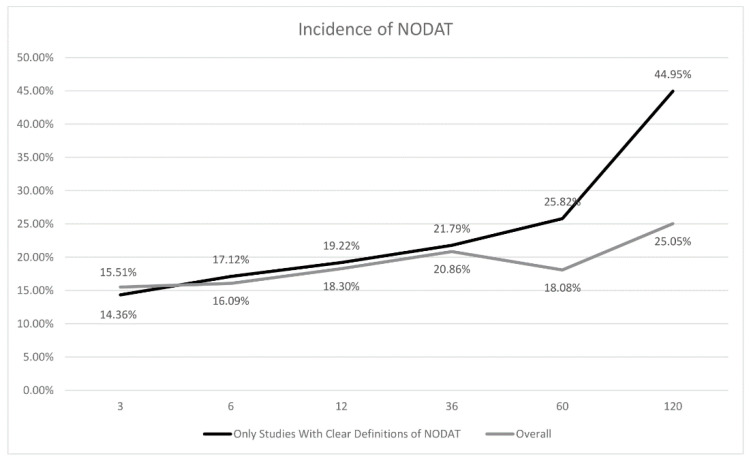
Incidence of New-Onset Diabetes after Transplantation (NODAT) at Various Timepoints.

A sensitivity analysis was conducted where only articles using the WHO and ADA definitions of NODAT were included in the analysis (Figure 5). The incidence of NODAT was relatively similar at three-months (14.4%; CI:11.1% to 18.4%), six-months (17.1%; CI:12.7% to 22.7%), one-year (19.2%; CI:15.3% to 23.9%), but higher at three-year (21.8%; CI:11.2% to 38.1%), five-year (25.8%; CI:10.9% to 49.7%), and ten-year (45.0%; CI:30.6% to 60.2%) timepoints when compared to overall analysis (Table 2).

After the sensitivity analysis, included articles were then subgrouped according to the predominant ethnicity of the patients and analyzed. No significant differences in the incidence of NODAT was found for all timepoints (Table 2). However, other than the three-months timepoint, studies from predominantly Asian populations had a higher incidence of NODAT as compared to studies from predominantly Western populations for all other timepoints (Table 2).

## 4. Discussion

NODAT has been observed to have an adverse effect on patient survival and graft survival and an increased incidence of infectious complications, where patients with NODAT had higher rates of postoperative bacterial infections and lower survival rates compared to patients without NODAT [5]. In addition, patients with NODAT are more likely to experience an acute rejection episode [7,56]. The results of this meta-analysis suggested that the overall incidence of NODAT after liver transplant at three-months, six-months, one-year, three-year, five-year, and ten-year timepoints ranged between 15% to 25%. When considering only articles which had clear definitions of NODAT, the incidence of NODAT increased, and it ranged between 15% to 45%. The incidence was comparable, if not higher than that of NODAT after renal transplant which ranged between 4% to 25% [12]. It is also higher in comparison to a previous meta-analysis conducted by Heisel et al., which ranged between 7.7% to 18.2% [19].

Evidence for the effect of ethnicity on the incidence of NODAT has been conflicting [10,11,14,36]. This meta-analysis found that studies with patients predominantly from Asian populations had a non-significant increase in incidence of NODAT compared to articles with patients predominantly from Western populations (Table 2). Previous studies have suggested that ethnicity may potentially affect the incidence of NODAT [10]. However, the influence of ethnicity on NODAT has yet to be confirmed [10], and these studies had small sample sizes in their analysis. Furthermore, there was high heterogeneity across the included studies, and current evidence on the issue is insufficient. More multicentric studies and trials would be needed to assess whether ethnicity could affect incidence of NODAT [16], and further studies aimed at explaining the sources of heterogeneity between the studies should be conducted.

In the overall analysis, incidence of NODAT at five-year was noted to have a decrease in the incidence compared to three-year incidence (Figure 5) likely due to the result of variable definitions. However, after sensitivity analysis for articles using WHO/ADA guidelines [9], a more temporal relationship with estimates of 25.82% and 44.95% for five-year and ten-year timepoints respectively was found. Our analysis also found that the incidence of NODAT was much higher in the three-year, five-year, and ten-year timepoints as compared to those one-year or less after the sensitivity analysis (Figure 5). This could be attributed to the lack of clear definitions of NODAT, and it has been an issue for the study of this condition [9,10,12], leading to a variation in reported incidence of this condition. Additionally, a short observational period for NODAT (<1 year) can also lead to an underestimation of the true incidence of NODAT [9], and many patients developed the condition many years after transplant [9,10,12]. Thus, reporting of incidence of NODAT should include timepoints longer than one-year to better understand the true incidence of NODAT.

While the importance of reporting the incidence of NODAT after one-year is important, we do note that the development of NODAT at five- and ten-years post-transplant may be significantly different compared to those at six-month or one-year post-transplant. Those developed at a later timing may be affected more by lifestyle habits such as diet, smoking and physical activity as well as the development of other comorbidities such as hyperlipidaemia and hypertension [57]. In addition, our analysis may be affected by survival bias, where patients who survive to ten-year posttransplant may be patients with lower BMI, of more benign disease and of a younger age, factors which can affect the development of NODAT (Supplementary Material 2) [58].

In addition, well known risk factors such as the use of living donor liver transplant (LDLT) compared to dead donor liver transplant (DDLT) recipients, socio-cultural, diet and lifestyle and varied immunosuppressive agents used may have confounded our analyses. We were, however, unable to exclude or control for all these factors in our analyses. A list of risk factors, both modifiable and non-modifiable, involved in NODAT development have been compiled in Supplementary Material 2. LDLT recipients have been noted to have lower incidences and lowered risk for NODAT compared to DDLT recipients [11,14,36]. Differences in the type of liver donors have been noted between various countries, which could affect the incidence of NODAT [59]. Another reason may be attributed to the varied diabetogenic effect of immunosuppressive agents and greater risk of diabetes due to cultural and lifestyle differences [9]. There is also variance in the immunosuppressive strategies and protocols in the different countries, with some using steroid-free regimens [60], and mTOR-I (mammalian target of rapamycin inhibitors) [12,61,62], which affects the incidence of NODAT. Lastly, variations in the indications for liver transplant were noted between countries, and this could have affected the incidence of NODAT. Asians were noted to be more commonly indicated for liver transplant due to hepatitis B virus (HBV), and HCC while patients from Western populations were more likely to need liver transplant due to HCV and alcoholic liver disease related liver failures [63].

Considering the high incidence of NODAT, there has been increasing calls for more stringent surveillance and management of NODAT. The International Consensus Meeting on Post-transplantation Diabetes Mellitus in 2014 suggested that besides the increased screening recommendations in the first year post-transplant, and annual screens thereafter, an additional pre-transplant baseline evaluation should be done to evaluate the risk of developing NODAT [9,64]. This includes a complete medical, family, and glucose history, as well as additional factors such as body weight and HCV status [9,64]. Individualisation of immunosuppressive treatment, to better balance the individual risks between transplant rejection and increasing their risk of developing NODAT, can also be implemented for patients to maximise their benefits from the treatment [9]. Additionally, screening for NODAT using postprandial glycemia and (glycated haemoglobin) HbA1c are also recommended to better streamline investigations for NODAT [64]. A stepwise approach for the management of NODAT is also recommended, where guidance for lifestyle modifications and exercise to reduce the risk of developing NODAT after liver transplantation, followed by additional opinion-based guidance for pharmacological therapy [64]. These recommendations would allow for early identification and investigations of high-risk individuals for developing NODAT, reducing the morbidity and mortality of NODAT through early intervention [64].

### Strength and Limitations

To the best of our knowledge, this is the most detailed meta-analysis analysing the incidence of NODAT after liver transplantation in various timepoints. Previous studies systematically reported the prevalence of NODAT [18], post-transplant metabolic diseases [65], compared the incidence of NODAT without considering the specific timepoints or were conducted before the introduction of the WHO and ADA guidelines [12,19]. Additionally, this meta-analysis compared the possible difference in Asian and European incidence of NODAT and suggests further analysis to confirm these findings. However, there are several limitations in this meta-analysis. Firstly, only English articles were included in this paper, which may limit the generalisability of the incidence of NODAT. Secondly, there were few articles examining the incidence of NODAT aside from the one-year timepoint (Table 2), and this may have led to a lack of generalisability of our findings and possible biasness of the results found. Additionally, we were unable to analyse the differences in steroid dosages due to tapered immunosuppressive regimen, which is individualised to each patient. Next, significant funnel plot asymmetry was found, and many studies were not located within the funnel plot. This which suggests that there is the presence of publication bias or heterogeneity in the current literature pool, which would have affected the results of the analysis. Lastly, most of the pooled estimates had a high I^2^ value (>75%), suggesting that there is a large heterogeneity in our summary estimates. However, the use of I^2^ for assessing statistical heterogeneity in studies with large sample sizes, such as prevalence or incidence meta-analyses, is debatable, and previous studies have shown that overreliance on I^2^ to assess heterogeneity may be misleading [26,66].

## 5. Conclusions

In summary, this meta-analysis shows that NODAT is common after liver transplant with one quarter of individuals developing NODAT 10 years after transplant. The incidence of NODAT increases with years after liver transplantation, suggesting there are potentially reversible factors that could exacerbate NODAT. Further studies exploring the impact of ethnicity and genetics on the incidence of NODAT, and clinical trials examining specific pharmacotherapies for NODAT in liver transplant patients are also needed.

## Figures and Tables

**Table 1:** Summary of Included Articles.

Author	Year	Sample Size	Gender (M)	Age	BMI	Immunosuppressive Regimen	NOS Score
Jain AB et al. [31]	1999	121	0.56	46.3 ± 12.3	-	Tacrolimus, Cyclosporine, Prednisone, Azathioprine	4
Driscoll et al. [32]	2006	115	0.71	48.5 ± 10.9	27.4 ± 6.1	Tacrolimus, Cyclosporine, Sirolimus, MMF, Azathioprine	6
Saliba et al. [42]	2007	211	0.71	52.7 ± 9.8	25.3 ± 4.68	Tacrolimus, Steroids	6
Oufroukhi et al. [41]	2008	141	0.67	52.6 ± 10	24.7 ± 5	Tacrolimus, Steroids, MMF	5
Zhao et al. [38]	2009	84	0.83	42.5 ± 9.1	22.2 ± 3.2	Tacrolimus, Cyclosporine	4
Carey et al. [36]	2011	225	0.71	51.7 ± 9.8	28.1 ± 5.2	Tacrolimus, Cyclosporine, Sirolimus	6
Honda et al. [45]	2013	161	0.47	47.2 ± 12.9	22.6 ± 3.8	Tacrolimus, Cyclosporine, Steroids, MMF	5
Rubin et al. [48]	2013	158	0.67	44.75 ± 9.60	25.25 ± 3.39	Cyclosporine, Tacrolimus, Azathioprine, Steroids	6
Cho et al. [53]	2014	364	0.69	49.98 ± 9.18	23.62 ± 3.23	Steroids	6
Parvizi et al. [52]	2014	350	0.58	39.24 ± 16.24	22.70 ± 4.63	Tacrolimus, Mycophenolate, Prednisone	4
Varghese et al. [44]	2014	32	0.90	44.3 ± 12.4	-	Tacrolimus, MMF	4
Younossi et al. [34]	2014	18,571	0.75	54.04 ± 7.37	27.98 ± 5.45	Tacrolimus, MMF, Steroids	5
Cuervas-Mons et al. [47]	2015	117	0.79	55.60 ± 8.31	27.77 ± 4.48	Tacrolimus	4
Gebhardt et al. [51]	2015	81	0.70	55.10 ± 10.67	28.92 ± 6.05	Tacrolimus, Prednisone	4
Hartog et al. [55]	2015	430	0.8	48.25 ± 9.23	26.4 ± 5.1	Tacrolimus, Steroids	4
Stepanova et al. [35]	2015	17,184	0.59	51.82 ± 12.54	27.27 ± 5.87	Tacrolimus, Steroids, MMF	6
Liu et al. [54]	2016	2248	0.69	43.95 ± 19.14	-	Tacrolimus, Cyclosporine, MMF, Rapamune, Everolimus	5
Li et al. [13]	2016	18,741	0.67	53.62 ± 10.36	28.11 ± 5.79	Tacrolimus, Steroids, MMF	6
Ling et al. [6]	2016	10,204	0.83	48.20 ± 10.01	22.79 ± 2.91	Tacrolimus, Cyclosporine, Corticosteroids	5
Saliba et al. [40]	2016	180	0.81	54.25 ± 8.42	-	MMF	6
Song et al. [37]	2016	528	0.85	44.93 ± 9.41	-	Tacrolimus, Corticosteroids, MMF	5
Yagi et al. [46]	2016	175	0.50	51 ± 11	23.8 ± 0.3	Tacrolimus, MMF	5
Cen et al. [39]	2017	256	0.84	47.92 ± 7.34	22.53 ± 3.02	Tacrolimus, MMF, Steroids	4
Xue et al. [15]	2017	763	0.85	48.78 ± 10.23	23.06 ± 2.4	Tacrolimus, MMF, Corticosteroids	4
Lieber et al. [33]	2019	415	0.68	54.38	28.87	Tacrolimus, Mycophenolate, Sirolimus	5
Aravinthan et al. [49]	2019	2209	0.67	53.67 ± 9.64	26.67 ± 5.19	Tacrolimus, Cyclosporine, Sirolimus, Prednisone, Mycophenolate	6
Oommen et al. [43]	2020	51	-	45.6 ± 9.6	24.52 ± 4.63	Tacrolimus, Glucocorticoids, Azathioprine, Mycophenolate	4
Hashim et al. [50]	2020	100	0.91	52 ± 7.7	27.2 ± 4.4	Tacrolimus, Cyclosporine, mTOR, Steroids	5

“-“—Information was not available in the articles; All numbers are presented in mean ± standard deviation unless stated otherwise. BMI – Body Mass Index, DM—Diabetes Mellitus, M – Male, MMF—Mycophenolate Mofetil, mTOR—mammalian target of rapamycin, NOS—Newcastle Ottowa Scale.

**Table 2:** Incidence of New-Onset Diabetes after Transplantation (NODAT) after Liver Transplantation.

Study Period	Total Papers	Total Sample Size	Pooled Incidence	Total Sample Size (after Sensitivity Analysis)	Pooled Incidence	Incidence of NODAT from Studies from Predominantly Western Populations	Incidence of NODAT from Studies from Predominantly Asian Populations	*p*-Value
3 months	7	11226	15.51% (CI:11.47% to 20.65%)	11367	14.36% (CI:11.09% to 18.38%)	14.65% (CI:10.81% to 19.54%)	13.97% (CI: 9.65% to 19.81%)	0.8424
6 months	10	46203	16.09% (CI:10.89% to 23.12%)	11291	17.12% (CI:12.68% to 22.70%)	15.87% (CI:10.07% to 24.11%)	17.83% (CI:12.08% to 25.51%)	0.6926
1 year	24	65845	18.30% (CI:14.78% to 22.44%)	64298	19.22% (CI:15.30% to 23.88%)	16.39% (CI:12.83% to 20.29%)	20.04% (CI:15.51% to 25.00%)	0.2318
3 years	10	40882	20.86% (CI:12.95% to 31.84%)	20711	21.79% (CI:11.19% to 38.12%)	14.19% (CI:1.62% to 62.46%)	26.35% (CI:18.44% to 36.16%)	0.5204
5 years	10	35680	18.08% (CI:10.25% to 29.90%)	20318	25.82% (CI:10.90% to 49.73%)	20.77% (CI: 2.88% to 69.87%)	30.94% (CI:16.61% to 50.20%)	0.6516
10 years	4	3697	25.05% (CI:11.17% to 47.05%)	1291	44.95% (CI:30.56% to 60.24%)	-	-	-

“-”—data not available; CI: Confidence Interval.

## Data Availability

The data and articles presented in this study are openly available in Medline and Embase.

## Supplementary Materials

The following are available online at https://www.mdpi.com/2077-0383/10/5/1045/s1; Supplementary Material 1: Search Strategy, Supplementary Material 2: Modifiable and non-modifiable factors implicated in NODAT, Supplementary Material 3: Funnel plot of incidence of NODAT at 6-months after Liver Transplantation, Supplementary Material 4: Funnel plot of incidence of NODAT at 3-years after Liver Transplantation, Supplementary Material 5: Funnel plot of incidence of NODAT at 5-years after Liver Transplantation.
