# Supplementary material for: A Time-Based Meta-Analysis on the Incidence of New Onset Diabetes after Liver Transplantation
Source: J Clin Med. 2021 Mar 3;10(5):1045. doi: 10.3390/jcm10051045 (PMC7959476; doi:10.3390/jcm10051045)

Supplementary Material 1: Search Strategy

1. exp epidemiologic studies/ or exp epidemiology/ or epidemio*.tw. or exp prevalence/ or prevalence.tw. or exp incidence/ or incidence.tw. or occurrence.tw.
2. (nodat or nodalt or nodm or diabet* or PLTDM).tw.
3. ((liver* or hepat*) adj3 (transplan* or graft*)).tw. or exp Liver Transplantation/
4. 1 AND 2 AND 3

Supplementary Material 2: Modifiable and non-modifiable factors implicated in NODAT.

Factor (host)	Risk	Ratio*	Factor (modifiable)	Risk	Ratio*
Older age(1)	Increased	1.24 (HR)	Corticosteroid use(1)	Increased	1.59 (HR)
Male gender(2)	Increased	1.53 (OR)	Tacrolimus use(2)	Increased	1.34 (OR)
High BMI(2)	Increased	1.19 (OR)	Induction agent(1)	Decreased	0.82 (HR)
Subcutaneous fat(3)	Increased	1.43 (OR)	CMV infection(4)	Increased	2.51 (HR)
Family history of DM(2)	Increased	1.69 (OR)	Magnesium levels(4)	Decreased	0.66 (HR)
Ethnicity (Hispanics)(5)	Increased	2.30 (OR)	Statin use(6)	Increased	2.32 (HR)
Ethnicity (African American)(1)	Increased	1.15 (HR)	Donor age > 60(7)	Increased	1.21 (HR)
Underlying disease (HCV)(2)	Increased	2.68 (OR)	Donor gender (male)(8)	Increased	1.98 (OR)
Underlying disease (NASH)(9)	Increased	1.21 (HR)	Donor liver steatosis(10)	Increased	1.77 (HR)
ICU stay > 15 days(8)	Increased	2.43 (OR)	Cold ischemia time > 9 hours(8)	Increased	1.16 (OR)

*Ratio represents odds ratios (OR) or hazard ratios (HR) reported in individual studies.

BMI – Body Mass Index, CMV – Cytomegalovirus, DM – Diabetes Mellitus, HCV – Hepatitis C Virus, ICU – Intensive Care Unit, NASH – Non-alcoholic Steatohepatitis

REFERENCES

1. Kuo HT, Sampaio MS, Ye X, Reddy P, Martin P, Bunnapradist S. Risk factors for new-onset diabetes mellitus in adult liver transplant recipients, an analysis of the Organ Procurement and Transplant Network/United Network for Organ Sharing database. *Transplantation.* 2010;89(9):1134-1140.
2. Li DW, Lu TF, Hua XW, Dai HJ, Cui XL, Zhang JJ, et al. Risk factors for new onset diabetes mellitus after liver transplantation: A meta-analysis. *World J Gastroenterol.* 2015;21(20):6329-6340.
3. Vaughn VM, Cron DC, Terjimanian MN, Gala ZS, Wang SC, Su GL, et al. Analytic morphomics identifies predictors of new-onset diabetes after liver transplantation. *Clin Transplant.* 2015;29(5):458-464.
4. Van Laecke S, Desideri F, Geerts A, Van Vlierberghe H, Berrevoet F, Rogiers X, et al. Hypomagnesemia and the risk of new-onset diabetes after liver transplantation. *Liver Transpl.* 2010;16(11):1278-1287.
5. Couto CA, Gelape CL, Doycheva IB, Kish JK, Martin P, Levy C. Ethnicity predicts metabolic syndrome after liver transplant. *Hepatol Int.* 2013;7(2):741-748.
6. Cho Y, Lee MJ, Choe EY, Jung CH, Joo DJ, Kim MS, et al. Statin therapy is associated with the development of new-onset diabetes after transplantation in liver recipients with high fasting plasma glucose levels. *Liver Transpl.* 2014;20(5):557-563.
7. Yadav AD, Chang YH, Aqel BA, Byrne TJ, Chakkera HA, Douglas DD, et al. New Onset Diabetes Mellitus in Living Donor versus Deceased Donor Liver Transplant Recipients: Analysis of the UNOS/OPTN Database. *J Transplant.* 2013;2013:269096.
8. Ling Q, Xu X, Xie H, Wang K, Xiang P, Zhuang R, et al. New-onset diabetes after liver transplantation: a national report from China Liver Transplant Registry. *Liver Int.* 2016;36(5):705-712.
9. Li Z, Sun F, Hu Z, Xiang J, Zhou J, Yan S, et al. New-Onset Diabetes Mellitus in Liver Transplant Recipients With Hepatitis C: Analysis of the National Database. *Transplant Proc.* 2016;48(1):138-144.
10. Xue M, Lv C, Chen X, Liang J, Zhao C, Zhang Y, et al. Donor liver steatosis: A risk factor for early new-onset diabetes after liver transplantation. *J Diabetes Investig.* 2017;8(2):181-187.

Supplementary Material 3: Funnel plot of incidence of NODAT at 6-months after Liver Transplantation

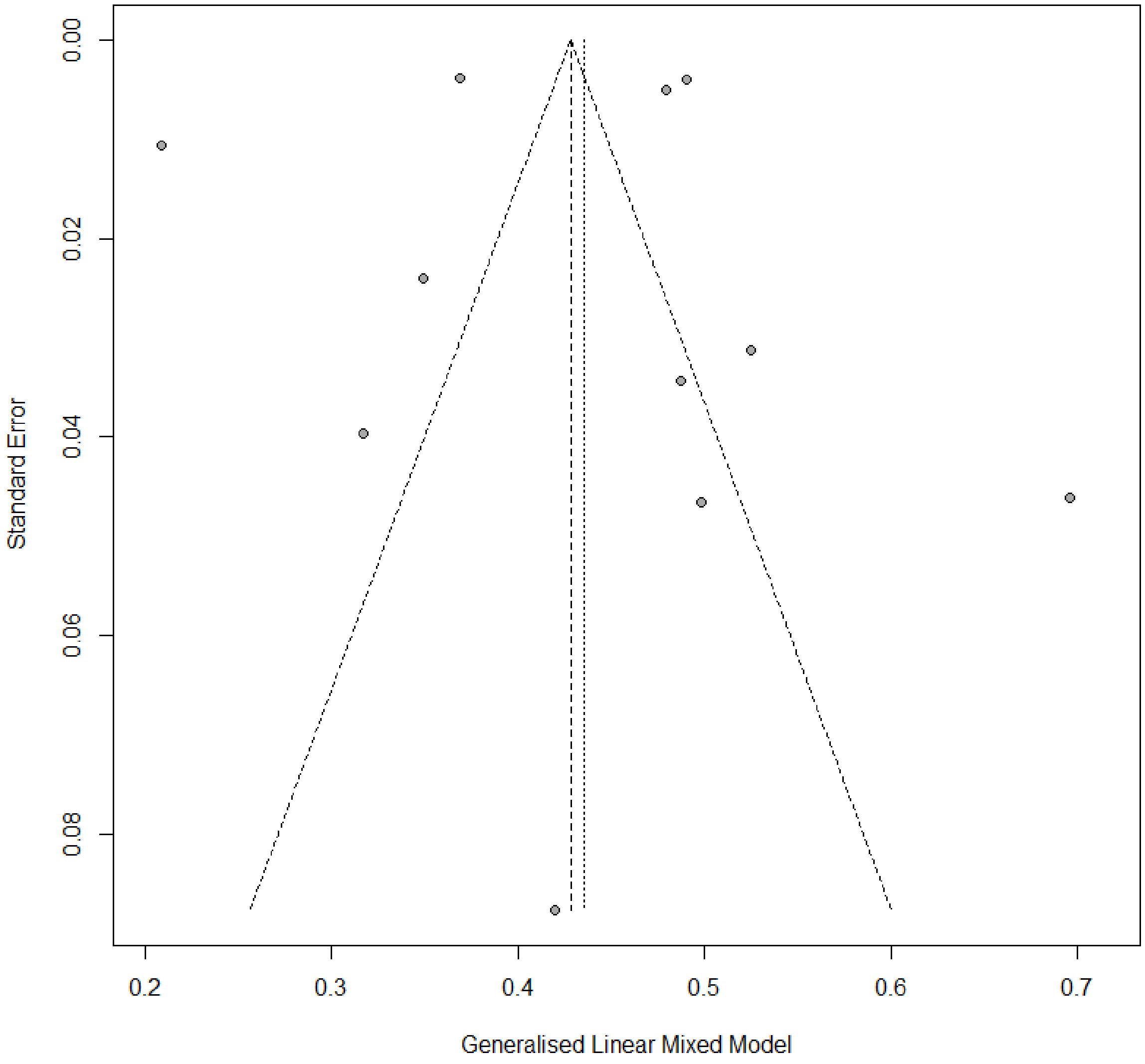

Supplementary Material 4: Funnel plot of incidence of NODAT at 3-years after Liver Transplantation

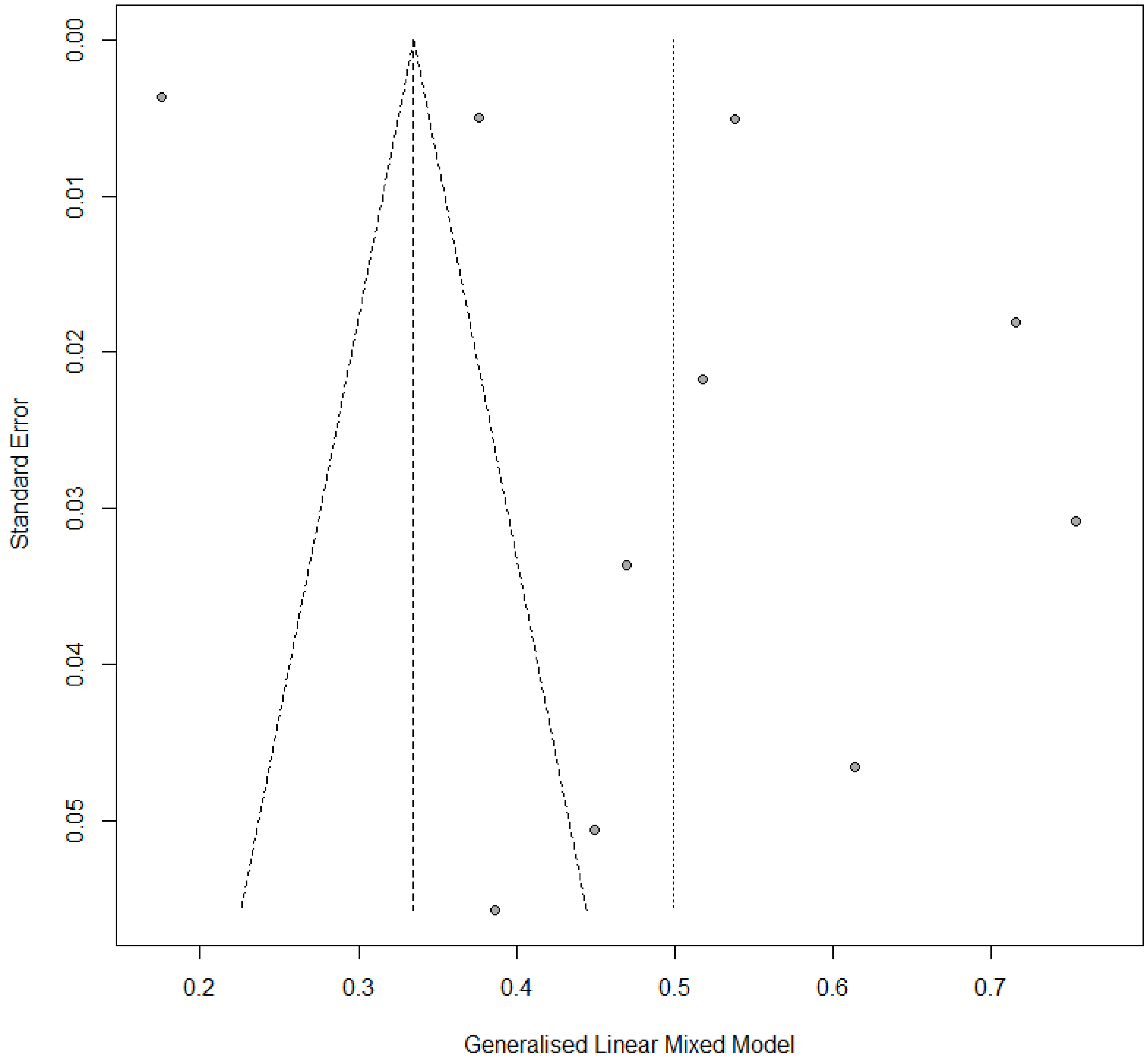

Supplementary Material 5: Funnel plot of incidence of NODAT at 5-years after Liver Transplantation

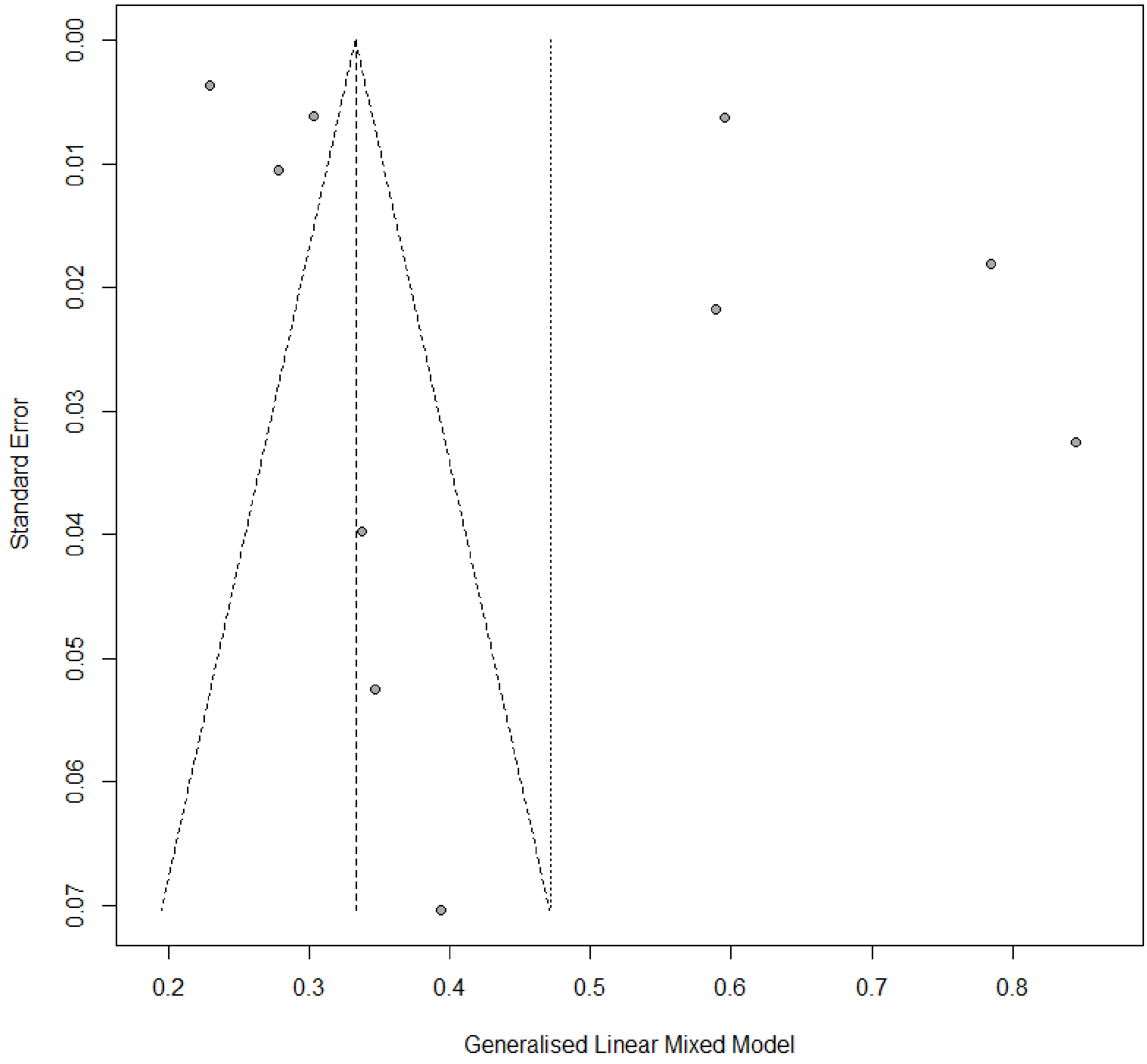

Supplement: Supplementary file 1 [file jcm-10-01045-s001.pdf]
